# Values and economic performance across European welfare state regimes: Direct and indirect effects through social capital, human capital and managerial skills

**DOI:** 10.1371/journal.pone.0298667

**Published:** 2024-02-23

**Authors:** Katarzyna Growiec, Marcin Czupryna, Jakub Growiec

**Affiliations:** 1 SWPS University, Warsaw, Poland; 2 Cracow University of Economics, Kraków, Poland; 3 SGH Warsaw School of Economics, Warsaw, Poland; Cavendish University / Kyambogo University, UGANDA

## Abstract

The values that people hold are linked to their economic performance. These links can be either direct or indirect, operating through moderating variables such as social network participation, interpersonal trust, trust in institutions, human capital, managerial skills and hours worked. In this paper these effects are studied using structural equation modelling (SEM) methodology applied to European Social Survey data from 28 European countries in 2018. Schwartz classification of values is used, distinguishing between Self-Enhancement (Power, Achievement), Openness to Change (Self-Direction), Conservation (Tradition, Security, Conformity) and Self-Transcendence (Universalism, Benevolence) values. It is found that Power has the strongest positive direct effect on economic performance, further strengthened by a positive indirect structural effect through hours worked. Self-Direction is indirectly positively linked to economic performance through higher managerial skills and hours worked. Tradition has a strong negative direct effect on economic performance. Security is indirectly negatively linked with economic performance, owing to its negative effects on interpersonal trust, management skills and hours worked. Some of the identified effects are context-dependent and vary across European welfare state regimes. For example, Power is statistically significantly linked to economic performance only in the liberal and conservative regime. Values promoted by respective welfare state regimes are not necessarily associated with higher incomes within those regimes, e.g., Tradition and Security values promoted in the conservative and Mediterranean regime are associated with lower incomes. These findings may lead to a range of policy implications, particularly in relation to the policies on immigration, demographics, the labor market, and work-life balance. Unfortunately, due to the cross-sectional character of the dataset, causal relations among the variables of interest could not be identified.

## 1. Introduction

Researchers have struggled for centuries with the question: what underlies the variation in individuals’ economic performance, as measured by their wages or labour productivity? With today’s knowledge, the answer should probably point at human capital differences, reflected in people’s education, work experience and health, people’s social and managerial skills, and their social capital: the resources embedded in their social networks. The answer could also include capital augmentation of the workplace and characteristics of each individual’s employer. In a cross-country setting, the answer could also involve the country’s institutions and social norms. However, the sources of variation in individuals’ economic performance can also be sought deeper, in the values that are important for people and which underlie their actions. Already Adam Smith discussed a variety “moral sentiments” and, for example, argued for individualism and self-interest as important drivers of economic performance [1]. In turn, Weber identified the Protestant work ethic as the cause for relatively strong economic performance of Protestant nations in the 19th century [2]. However, despite these early contributions, the topic has never grown into a systematic research program. In this paper, we provide additional insights to the relationship between people’s values and their economic performance, and investigate what would be the channels of their influence: through which actions do values impact performance? We aim to address these research questions using the definition of values proposed by Schwartz [3, 4], broadly accepted in the psychological literature but largely neglected by economists.

As Shalom Schwartz put it: “A value is a (1) belief (2) pertaining to desirable end states or modes of conduct, that (3) transcends specific situations, (4) guides selection or evaluation of behaviour, people, and events, and (5) is ordered by importance relative to other values to form a system of value priorities” ([4], p. 20); see also [3, 5–9]). As many classical works have acknowledged, values are an important driver of economic performance [1, 2, 10]. However, in recent economic and sociological literature values have received surprisingly little attention [11]. This may be because in survey data the correlation between values and action is generally weak: it never exceeds 0.2 across different cultures and behaviours [12]. On the one hand, this is to be expected: individual behaviour is largely determined by situational factors, and hence the direct impact of values on individual behaviour must be limited. On the other hand, though, values can determine individual behaviour also indirectly, e.g., through social schemes or social institutions. For this reason, there has been a recent revival of interest in values, seen as having both a direct and indirect impact on action [11, 13–15].

The most influential survey questionnaire for measuring values was introduced by Schwartz [3]. It was subsequently extended [16] and included in the European Social Survey, a cross-national survey programme. According to Schwartz [16], values are related to people’s attitudes, norms and expectations in the economic sphere, such as the importance of work in one’s life (“work centrality”), societal norms about working (whether people are entitled or obliged to work) and the goals or rewards that people expect from their work. The latter might be intrinsic (like personal growth, autonomy and creativity), extrinsic (pay and security), social (having contacts with other people) or related to power (gain in power and authority). These attitudes, norms and expectations have, in turn, an impact on people’s actions and economic performance.

The objective of this paper is to identify direct and indirect structural effects of values on individuals’ economic performance–represented by their net pay–across 28 European countries. We measure values with Schwartz’s Portrait Values Questionnaire (PVQ; [17]), which groups them into 10 domains: Power, Achievement, Hedonism, Stimulation, Self-Direction, Universalism, Benevolence, Tradition, Conformity, and Security. The considered channels of possible indirect impact include people’s social network participation, interpersonal trust, trust in institutions, human capital, managerial skills and hours worked. Our analysis uses structural equation modelling (SEM), carried out on the basis of individual cross-sectional data from the ninth wave of the European Social Survey (2018). Our analysis fills an important gap in the literature: the link between values as defined by Schwartz [3] and individuals’ economic performance (e.g., net pay) have not been systematically studied yet. In particular, to our best knowledge there have been no studies of direct and indirect structural effects of values on economic performance.

We supplement a Europe-wide investigation with an additional study where we split the European sample into five subsamples. Namely, as Europe is not homogeneous in terms of culture and values–individual-level variation in values is convoluted with variation at national and regional levels–we also compare whether our results vary across European welfare state regimes [18, 19]. We expect to obtain at least some differences here, because each welfare state regime provides a different cultural context that offers different models for action. We consider five European welfare state regimes [20]: liberal, conservative, socio-democratic (Nordic), Mediterranean and post-socialist.

Identification of differentiated relationships between values and economic performance across European welfare state regimes constitutes another contribution of this paper to the literature. Its importance follows from the fact that it is not the norms themselves, but the interaction of norms and the context that matters for individuals’ actions and outcomes [21]. Individuals’ economic activity takes place in social structures and is shaped by social networks. In this regard, welfare state regimes provide a richer characterization of the diversity of social contexts across Europe than alternative specifications of cultural dimensions present in the literature, such as the tightness-looseness divide, focusing on the number of social norms and tolerance for deviant behaviour [22, 23].

## 2. Literature review and hypotheses

### 2.1. Values in social science

The first prominent sociological theory of values was Talcott Parsons’ structural functionalism [24–27]. Parsons argued that societies instil values in their members, which in turn shape their normative expectations, establish standards of evaluation of their behaviour, and operate as goals for action. These societally shared sets of values help maintain social order and help individuals select their goals in a conscious and rational way [28, 29].

The most advanced programme of measuring values to date was introduced and conducted by Shalom Schwartz. He proposed a 10-value structure that overcame earlier theoretical pitfalls [3, 4]. According to Schwartz’s theory, values and action must be measured separately, as a prerequisite for testing the structure of values in real life. Schwartz also proposed a theory of value content and structure. As he put it: “I define *values* as desirable trans-situational goals, varying in importance, that serve as guiding principles in the life of a person or other social entity. Implicit in this definition of values as goals is that (1) they serve the interests of some social entity, (2) they can motivate action–giving it direction and emotional intensity, (3) they function as standards for judging and justifying action, and (4) they are acquired both through socialization to dominant group values and through the unique learning experiences of individuals” ([4], p. 21). According to the social cognitive theory, environment provides a source of possible patterns of behaviour and information about their efficacy and social approval [30, 31]. Values dominant in a certain society (or welfare state regime) are sources of goals that guide people’s behaviour, create standards of behaviour evaluation and, as a consequence, reinforce behaviour that leads to conformity with the socially approved goals.

Schwartz’s typology of values consists of oppositional pairs between competing value types [3, 4]: Power vs. Universalism; Achievement vs. Benevolence; Hedonism and Stimulation vs. Conformity and Tradition; Self-Direction vs. Security. An important novel feature of Schwartz’s theory is that “Values form a continuum of related motivations. It is this continuum that gives rise to the circular structure. The nature of the continuum is clarified by noting the shared motivational emphases of adjacent value types” ([4], p. 24). For example, Power and Achievement both emphasize social superiority and esteem. “Competing value types emanate in opposing directions from the centre; compatible types are in close proximity going around the circle” ([4], p. 24). We follow Schwartz’s typology of values closely in our empirical model.

Values are relatively stable during an individual’s lifetime [32]. They are central to the self and serve important psychological functions, e.g. motivational and normative. For this reason personal values are highly resistant to change [33]. However, some changes in personal values are nevertheless possible [5, 34], driven by external events along the life course or by self-chosen life transitions [35]. For example, people who experience autonomy at work may acquire Self-Direction and downgrade Security and Conformity [36]. Kiley and Vaisey’s panel data analysis confirms that values, similarly to attitudes or worldviews, are stable from the time of early socialization [37]. If a change in such individual dispositions is to happen, it typically occurs in people’s youth. Therefore, in the empirical model considered in this paper, we treat personal values as exogenous variables and analyse how they are linked to a variety of socio-economic circumstances, treated as endogenous variables in our model. Still, as our data are cross-sectional, we cannot verify whether these links represent causal relations.

### 2.2. Values and economic performance

Values, as defined by Schwartz, have been found to affect a variety of socio-economic outcomes in the literature, even if the studies have been scattered and did not form a systematic research program. For example, Jaén Figueroa and Liñán, based on data from Spain, identified links between values and entrepreneurial capital, conducive to flexibility and innovation [38]. Callaghan, in turn, found that economic performance among South African inner-city street traders was better among individuals with higher levels of Power and Hedonism values [39]. Primc et al. using European Social Survey data found that post-materialist values like Benevolence, Universalism and Self-Direction are important predictors of people’s pro-environmental behaviour [40].

Crucially for the current study, Schwartz [16] looked at the relation between cultural values and attitudes toward work also at the level of whole societies. (More recently, Ralston et al. compiled a large 50-country values dataset, focusing on values reported by business managers and professionals [41]). Schwartz claimed that societies which emphasize Mastery (corresponding to Achievement at the individual level) and Hierarchy (Power and Security; [4, 16, 34]) encourage individuals within that society to think of work as central to their lives ([16], p. 40). In contrast, when Affective Autonomy (Hedonism, Self-Direction, Stimulation at the individual level), Egalitarianism (Universalism and Benevolence), Harmony (Conformity) and Conservatism (Tradition and Security) prevail in a society, this reduces the perception of work as central to one’s life. Affective Autonomy promotes the pursuit of leisure. The importance of community in one’s life goes together with Egalitarian values, while the importance of family is promoted by Conservatism. When it comes to societal norms about working, namely whether people are entitled to it or obliged to do it, Schwartz [16] concludes that work is viewed as entitlement in cultures which value Autonomy and Egalitarianism. In turn, work as obligation goes together with Conservatism and Hierarchy, values which support the view that a person is an integral part of a larger collective with a certain role to play.

Finally, in what is perhaps the most important finding of the paper [16], Schwartz states that an extrinsic goal (e.g., pay) is most important for people in cultures which emphasise Conservatism and Hierarchy, and least important in cultures which promote Intellectual Autonomy. Autonomy values enhance the seeking of intrinsic rewards from work, e.g., personal growth, while Conservatism hampers it. Following Schwartz’s lead, Liñán and Fernandez-Serrano studied the role of national culture, as represented by the most salient Schwartz values, in shaping the level of economic development across European countries and determining the strength of the effect of entrepreneurship on incomes [42].

Against this background, the current study fills an important gap in the literature–the impact of values on individuals’ economic performance across 28 European countries, with the distinction between direct and indirect (structural) impacts. Specifically, in this study we make a connection between studies finding links between values that people hold and their social capital, human capital and managerial skills, and studies looking at the links between the latter variables and economic performance.

### 2.3. Latent variables: Social capital, human capital and managerial skills

In our study we argue that values may affect economic outcomes both directly and indirectly, by influencing such important determinants of people’s net pay as social capital (participation in social networks, interpersonal trust, trust in institutions), human capital and managerial skills.

In this regard, let us first disambiguate the concept of social capital. According to the OECD definition, social capital is a “network together with shared norms, values and understandings that facilitate cooperation within or among groups” [43]. Although the concept is generally ambiguous, following [44–47] we adopt a relatively narrow, focused definition that concentrates only on social networks and the immediately linked norms of reciprocity and trust. Some researchers (e.g., Woolcock [46]) see trust as an outcome of social capital (defined as networks and associated norms), while others view trust as a component of the shared values and norms which constitute social capital [43]. In the current study, we deliberately split “social capital” into (i) participation in social networks, (ii) interpersonal trust, and (iii) trust in institutions, as these dimensions do not necessarily go together and may be differently linked to economic performance [48–50].

#### 2.3.1. Participation in social networks

Social networks are comprised of a person’s social relationships; that is, “the set of people with whom an individual is directly involved” ([51], p. 2), such as “family members, friends, and acquaintances” ([52], p. 53). Social networks affect economic outcomes through their impact on the flow and quality of information [53]. The extent and structure of individuals’ social networks is also an important determinant of the magnitude of transaction costs they face, the possibility of implementing innovative but risky ideas in cooperation with others, and–in result–individuals’ willingness to cooperate and thrift [54–57]. Participation in social networks may also improve the non-pecuniary characteristics of the individual’s job, such as better career perspectives [58].

#### 2.3.2. Interpersonal trust

As Guiso, Sapienza and Zingales acknowledge, culture entered the economic discourse through the concept of trust [59]. Following the seminal contributions such as Banfield [60] or Putnam et al. [61], economists started to study the economic pay-off of interpersonal trust [62]. Fukuyama also found a positive link between interpersonal trust and economic performance [63]. Furthermore, it has also been argued that interpersonal trust increases the likelihood of becoming an entrepreneur and of being self-employed [59]. However, the links between trust and individual-level outcomes such as economic performance or well-being can be culture-dependent and generally tend to be stronger in individualistic societies [64].

#### 2.3.3. Human capital

People’s knowledge and skills, proxied by the number of years of education, are an important determinant of people’s earnings [65–67]. Furthermore, Coleman [68] emphasised the complementarity of social capital and human capital in achieving desirable economic outcomes; people with low skills or low levels of education are at higher risk of unemployment and social exclusion [69]. Therefore, in our model we include human capital as the next latent variable. Healy and Cote define human capital as “knowledge, skills, competences and attributes embodied in individuals that facilitate the creation of personal, social and economic well-being” ([43], p. 13).

However, human capital acquisition starts within the family and early childcare settings. For example, according to Bourdieu family background is extremely important when it comes to shaping attitudes towards learning, work habits and the aspirations of children [70, 71]. Therefore, we operationalize human capital in our model by the educational credentials of the respondents, but also instrumentalize it using the education of their mother and father [72].

#### 2.3.4. Links between latent variables

There are many links between our latent variables. Network participation and structure are important for the formation of interpersonal trust [73]. For instance, dense networks, compared to sparse networks with plentiful structural holes, lead to relatively greater conformity but less interpersonal trust [53, 74–76]. People taking a central position in a social network are also statistically more likely to be better educated and exhibit superior managerial skills compared to those in the periphery [77, 78]. Interpersonal trust and education are also related: increases in average education levels typically go together with increased levels of trust [43, 79]. However, this positive association may become negative in societies where social and political risks are widespread [80]. Finally, there are also links between trust and managerial skills. For instance, trusting others significantly increases the probability of becoming an entrepreneur [59].

#### 2.3.5. Links between values and latent variables

Kalish and Robins state that people who opt for dense social networks, closure, interpersonal trust, and the social support that they provide, tend to hold allocentric values like duty, obedience and security [81]. On the contrary, people who opt for loose social networks or the position of a broker in a social network tend to have an aggressive entrepreneurial personality [82, 83]. They prefer autonomy, risk-taking and change to the *status quo*, and may be relatively more aggressive and neurotic. This is congruent with Schwartz’s theory of values [4], where Self-Direction and Stimulation compete with Security, Conformity and Tradition. Greenberg, Haidt and Rodin, and Kadushin, however, emphasize that two human motivations that lie beneath these values, namely safety and effectance motivation, always cooccur: a person cannot reach out without a safe base of close, unconditional social ties. To be autonomous, an individual must first feel safe and supported [84–86]. Yet, “(…)one or another [motive] is likely to dominate conscious experience at any particular moment” ([84], p. 138).

Taking stock, the literature suggests that values are stable, resistant to change among adult people, and central to the self. They have impacts on individuals’ actions, including the ones in the social and economic sphere. These actions, in turn, culminate in the accumulation of capitals and skills, conducive to economic performance. This chain of findings from the literature suggests to use a structure where values are assumed exogenous, and actions are considered as latent variables, mediating the impact of values on economic performance. For example, it may be the case that those who value Power and Achievement earn more on average, but not because of holding these values per se, but because these values make them work harder, and longer hours than others.

### 2.4. European welfare state regimes

In our study we compare the effects of values on economic performance across European welfare state regimes. The relevance of this framework comes from the observation that norms as such do not matter for action; what matters is the interaction of norms and the context, emerging through social processes in social networks, limited by institutional ramifications [21].

Welfare state regimes provide the context within which individuals undertake their actions. They are the “key institutions in the structuring of class and the social order ([18], p. 55) because “[t]he welfare state is not just a mechanism that intervenes in, and possibly corrects, the structure of inequality; it is, in its own right, a system of stratification. It is an active force in the ordering of social relations” ([18], p. 23). Historically, they emerged and differentiated as a result of historical development. As Kääriäinen and Lehtonen sum up, “welfare state regimes differ in two basic dimensions: the degree of decommodification and the type of stratification/solidarity. Decommodification refers to the degree of freedom from market dependency, to the extent to which social-political benefits are social rights independent of markets (and family relations). Stratification refers to how extensive social political systems are, to the universality of benefits” ([20], p. 31).

We use the distinction between five European welfare state regimes [18, 87, 88]: liberal (GB, IE)–with minimum decommodification and a relatively narrow group of benefit recipients, conservative (AT, FR, CH, DE, BE, NL)–with intermediate levels of decommodification and strongly status-differentiating welfare programmes, socio-democratic (or Nordic; NO, FI, SE, DK)–highly decommodifying, with generous, broad-based social transfers, Mediterranean (ES, PT, IT, CY)–distinctively familist, with inconsistent, fragmented systems of welfare provision that may vary substantially in terms of generosity, and post-socialist (BG, CZ, EE, HR, HU, LT, LV, ME, PL, RS, SI, SK)–characterized by the experience of a shift from the broad-based Communist welfare state to a weakly decommodifying system with limited welfare services. Each welfare state regime provides a different cultural context that offers different models for action. These contexts may transform the impact of values on action.

As argued by Miles, welfare state regimes may mediate the link between values shape outcomes because “they can prime norms or other behavioural considerations that might override values or modify how they are expressed” ([11], p. 686). Welfare state regimes differ vastly when it comes to values promoted by them. According to Esping-Andersen “the classical liberal state attempted to grant the cash nexus a hegemonic role in the organization of social and economic life; the bottom line of liberal dogma was that the state had no proper reason for altering the stratification outcomes produced in the marketplace. They were just, because they mirrored effort, motivation, adeptness, and self-reliance” ([18], p. 62). Therefore the liberal welfare state regime promotes Achievement, Self-Direction but also Power as it leads to social stigma for those who fail on the market. In the conservative welfare state regime “what predominated was the preservation of status differentials; rights, therefore, were attached to class and status (…) But the corporatist regimes are also typically shaped by the Church, and hence strongly committed to the preservation of traditional familyhood” ([18], p. 27) with the state interfering only when family’s capacity fails. Therefore the conservative welfare state regime promotes Power, Security and Tradition. According to Esping-Andersen, the socio-democratic (Nordic) welfare state regime is an universalistic one striving to achieve equality of status. This equality is of “the highest standards, not an equality of minimal needs as was pursued elsewhere” ([18], p. 27). Therefore in this regime Universalism is the guiding principle. The post-socialist welfare state regimes present a mix of some aspects of all three welfare-state regimes described originally by Esping-Andersen and the Mediterranean welfare state regime is very close to conservative one but with stronger emphasis on Benevolence.

### 2.5. Hypotheses

Inspired by Schwartz’s results [16], we verify the relations between values and economic performance at the individual level. The dependent variable in our model is individual net pay, with values at the individual level serving as predictors of the level of income. First, we test the following hypothesis regarding our moderating variables:

H1. Individuals’ social capital (participation in social networks, interpersonal trust, trust in institutions), human capital, managerial skills and working hours per week are positively linked with income.

Next, we hypothesise that individuals whose values are focused around Power will be particularly motivated to have higher income, since extrinsic rewards from work are of importance to them. Individuals whose values are focused around Achievement should receive a relatively high income as well, as they think that work is the most important domain in their lives. As an implication of possessing such values, individuals may dedicate a lot of effort and time to work, and get a reward for that in terms of a relatively high salary. Therefore we will also ascertain whether they work more hours per week.

However, our aim is to go beyond direct effects and to understand the mechanisms underlying how Schwartz values may be indirectly related to economic performance. Therefore, we introduce latent variables in our model that can possibly help us identify the indirect structural effects. As latent variables, we use variables that are known to be related to economic performance: social capital (participation in social networks, interpersonal trust, trust in institutions), human capital, managerial skills and working hours per week. We hypothesise that specifically the value of Self-Direction may be positively related to interpersonal trust, human capital, managerial skills, and hours worked whereas the values of Tradition and Security may be negatively related to these variables [81, 82]. More broadly, we test the following hypotheses:

H2. Self-Enhancement values (Power, Achievement; we exclude Hedonism) are associated with higher income, both directly and indirectly through more hours worked.
H3. Openness to Change values (Self-Direction; we exclude Stimulation) are associated with higher income indirectly through interpersonal trust, human capital, managerial skills and hours worked.
H4. Conservation values (Tradition, Security, Conformity) are associated with lower income indirectly through interpersonal trust, human capital, managerial skills and hours worked.
H5. Self-Transcendence values (Universalism, Benevolence) are not associated with income.

As compared to Schwartz’s original typology [3], we exclude Hedonism and Stimulation from our study due to multicollinearity issues (explained later) as well as because these values are unlikely to be linked to work- and entrepreneurship-related activities of the individual.

Finally, we investigate whether the same values lead to the same outcomes across European welfare state regimes, or these results are context-dependent. We test the hypothesis that:

H6. Values promoted by European welfare state regimes (as listed by Esping-Andersen [18]: liberal regime–Power, Achievement, Self-Direction; conservative regime–Power, Security, Tradition; Nordic regime–Universalism; Mediterranean regime–Power, Security, Tradition, Benevolence) are associated with higher income in those regimes.

Specifically we expect that Power will be mostly associated with higher income in the liberal and conservative welfare state regimes because it is a guiding principle of the whole stratification system in those welfare state regimes. Action guided by this value might be socially more approved and justified. Therefore it might strengthen the link between Power and income. This might happen primarily through the indirect impact of Schwartz values on action [11].

## 3. Materials and methods

### 3.1. Dataset

Our data was obtained from the ninth (2018) wave of the European Social Survey (ESS), a cross-national, representative survey that includes measures of Schwartz’s value dimensions and various forms of self-reported behaviour. We only considered survey participants with pay (salary, wage) as the main source of income (based on the *fvgabc* variable), excluding those participants for whom pensions or social benefits were the main source of income. Observations with missing data (excluding the net income variable) were deleted, reducing the sample size from 26009 to 16435. Records with missing data were deleted, rather than imputed, from the dataset prior to analysis to avoid possible biases, with two exceptions. In the first case, it was possible to impute the missing information on some individuals’ net pay (*netinum*) based on the value of the variable *netilet* (for 2791 cases). The variable *netilet* contains the information on individuals’ net pay, coded into ten possible categories. The respective decile of the distribution for the *netinum* variable, for each country separately, was then taken as the value for each possible *netilet* category. In the second case, we substituted the missing information on the number of total hours worked with the country mean values (for 1965 cases).

### 3.2. Values

In the ESS, values are measured with the 21-item version of Schwartz’s Portrait Values Questionnaire (PVQ) [17]. In our study they were grouped in the following 10 domains: Power (social status and prestige, control or dominance over people and resources), Achievement (personal success through demonstrating competence according to social standards), Hedonism (pleasure and sensuous gratification for oneself), Stimulation (excitement, novelty, and challenge in life), Self-Direction (independent thought and action−choosing, creating, exploring), Universalism (understanding, appreciation, tolerance and protection for the welfare of all people and for nature), Benevolence (preservation and enhancement of the welfare of people with whom one is in frequent personal contact), Tradition (respect, commitment and acceptance of the customs and ideas that traditional culture or religion provide the self), Conformity (restraint of actions, inclinations, and impulses likely to upset or harm others and violate social expectations or norms), and Security (safety, harmony and stability of society, of relationships, and of self). These variables were additionally standardised at the country level by subtracting the respective country mean. Such transformation results from our general approach, where we take into account differences between individual values and the average value for a given country. In this way, the differences in the general economic development level between the countries considered in the analysis do not directly affect the results.

However, values adjacent on the circular structure of Schwartz’s core values are highly positively correlated, while values on the opposite sides of the circular structure are highly negatively correlated [4]. This feature leads to methodological concerns related to multicollinearity. To remedy this, following Schwartz’s suggestion we removed Hedonism and Stimulation–two Schwartz values indicated in the first step of the backwards stepwise regression procedure. The values of the Variance Inflation Factor [89] were all below 2 for the regressions with only eight remaining Schwartz values.

### 3.3. Latent variables

#### 3.3.1. Participation in social networks

The latent variable “participation in social networks” is comprised of the combined answers to three questions (we quote them precisely, following the ESS survey questionnaire, https://www.europeansocialsurvey.org/methodology/ess-methodology/source-questionnaire):

- *Sclmeet–*“How often do you meet socially with friends, relatives or work colleagues?” (1 –Never, 2 –Less than once a month, 3 –once a month, 4 –several times a month, 5 –once a week, 6 –several times a week, 7 –every day).
- *Inprdsc*–“How many people, if any, are there with whom you can discuss intimate and personal matters?” (0- None, 1–1, 2–2, 3–3, 4–4–6, 5–7–9, 6–10 or more).
- *Sclact*–“Compared to other people of your age, how often would you say you take part in social activities?” (1 –Much less than most, 2 –Less than most, 3 –About the same, 4 –More than most, 5 –Much more than most).

#### 3.3.2. Interpersonal trust

The latent variable “interpersonal trust” is comprised of the combined answers to three questions:

- *Ppltrst*–“Generally speaking, would you say that most people can be trusted, or that you can’t be too careful in dealing with people? Please tell me on a score of 0 to 10, where 0 means you can’t be too careful and 10 means that most people can be trusted.”
- *Pplfair*—“Do you think that most people would try to take advantage of you if they got the chance, or would they try to be fair? Please tell me on a score of 0 to 10, where 0 means most people try to take advantage of me and 10 means that most people try to be fair.”
- *Pplhlp*–“Would you say that most of the time people try to be helpful or that they are mostly looking out for themselves? Please tell me on a score of 0 to 10, where 0 means people mostly look out for themselves and 10 means that people mostly try to be helpful.”

#### 3.3.3. Trust in institutions

The latent variable “trust in institutions” is comprised of the combined answers to three questions:

- *Trstprl*–“Please tell me on a score of 0–10 how much you personally trust each of the institutions I read out. 0 means you do not trust an institution at all, and 10 means you have complete trust. Firstly… …[country]’s parliament?”
- *Trstlgl*–“Please tell me on a score of 0–10 how much you personally trust each of the institutions I read out. 0 means you do not trust an institution at all, and 10 means you have complete trust. (…)…the legal system?”
- *Trstplc*–“Please tell me on a score of 0–10 how much you personally trust each of the institutions I read out. 0 means you do not trust an institution at all, and 10 means you have complete trust. (…)…the police?”

#### 3.3.4. Human capital

The latent variable “human capital” is captured by the respondent’s number of years of schooling:

- *Eduyrs*–“Approximately how many years of education have you completed, whether full-time or part-time? Please report these in full-time equivalents and include compulsory years of schooling.”

#### 3.3.5. Managerial skills

The latent variable “managerial skills” is comprised of the combined answers to two questions:

- *Wkdcorga–*“Please say how much the management at your work allows/allowed you… …to decide how your own daily work is/was organised?” (0 –I have/had no influence, 10 –I have/had complete control).
- *Iorgact–*“Please say how much the management at your work allows/allowed you… …to influence policy decisions about the activities of the organization?” (0 –I have/had no influence, 10 –I have/had complete control).

#### 3.3.6. Working hours

In our model we also incorporate a measure of working hours per week (*wkhtot*). This variable is measured by a question: “Regardless of your basic or contracted hours, how many hours do/did you normally work a week (in your main job), including any paid or unpaid overtime.” Similarly, the variable *wkhct* measures the working time per week but with overtime excluded. Average hours worked at the country level are to a large extent determined by attitudes towards work and leisure [90]. The variable was normalized by dividing by the country-specific mean and taken in logs.

All variables, including the variables used for construction of latent variables as well as all variables representing Schwartz values were standardised by subtracting the mean of the respective variable for a given country.

#### 3.3.7. Additional control variables

We use age and age squared as additional control variables.

### 3.4. Dependent variable

The dependent variable in our model was measured by the question: “Your usual [weekly/monthly/annual] net [pay/pensions/social benefits]”, represented as the ESS variable *netinum*. It was normalized by dividing by the country-specific mean and taken in logs. The first transformation ensures that the variable has an approximately normal distribution. The second transformation makes it possible to compare data between different countries. The resulting variable can be interpreted as the percentage deviation from the mean net income in a given country.

We have also taken into account the analytical weight of a given observation in the entire population under consideration, using the ESS variable *anweight*, which “corrects for differential selection probabilities within each country as specified by sample design, for nonresponse, for noncoverage, and for sampling error related to the four post-stratification variables, and takes into account differences in population size across countries.” (from the ESS website https://www.europeansocialsurvey.org/methodology/ess_methodology/data_processing_archiving/weighting.html).

### 3.5. Methods

The analysis was carried out in two simultaneous steps. Firstly, six latent variables were constructed by means of confirmatory factor analysis [91]. Secondly, direct and indirect effect of values on net pay were estimated using structural equation modelling. The analysis was done in R using the *lavaan* package [92]. We applied the default WLSMV estimator, in which diagonally weighted least squares (DWLS) are used to estimate the model parameters, but robust standard errors are computed based on the full weight matrix. The estimator also uses a mean- and variance-adjusted test statistic [92]. This method is robust to the deviations from the multivariate normal distribution [91] but also addresses the problem of heteroskedasticity [93].

To identify the paths through which values are indirectly related to net pay, we formulated a structural equation model (SEM) with net income as our dependent variable. In this model we incorporated 6 latent variables: participation in social networks, interpersonal trust, trust in institutions, human capital, managerial skills and weekly hours worked. By identifying these indirect structural relations we show the paths through which Schwartz values are related to economic performance. (Recent applications of the SEM methodology to social variables include also, among others, [94, 95]).

**Fig 1:**
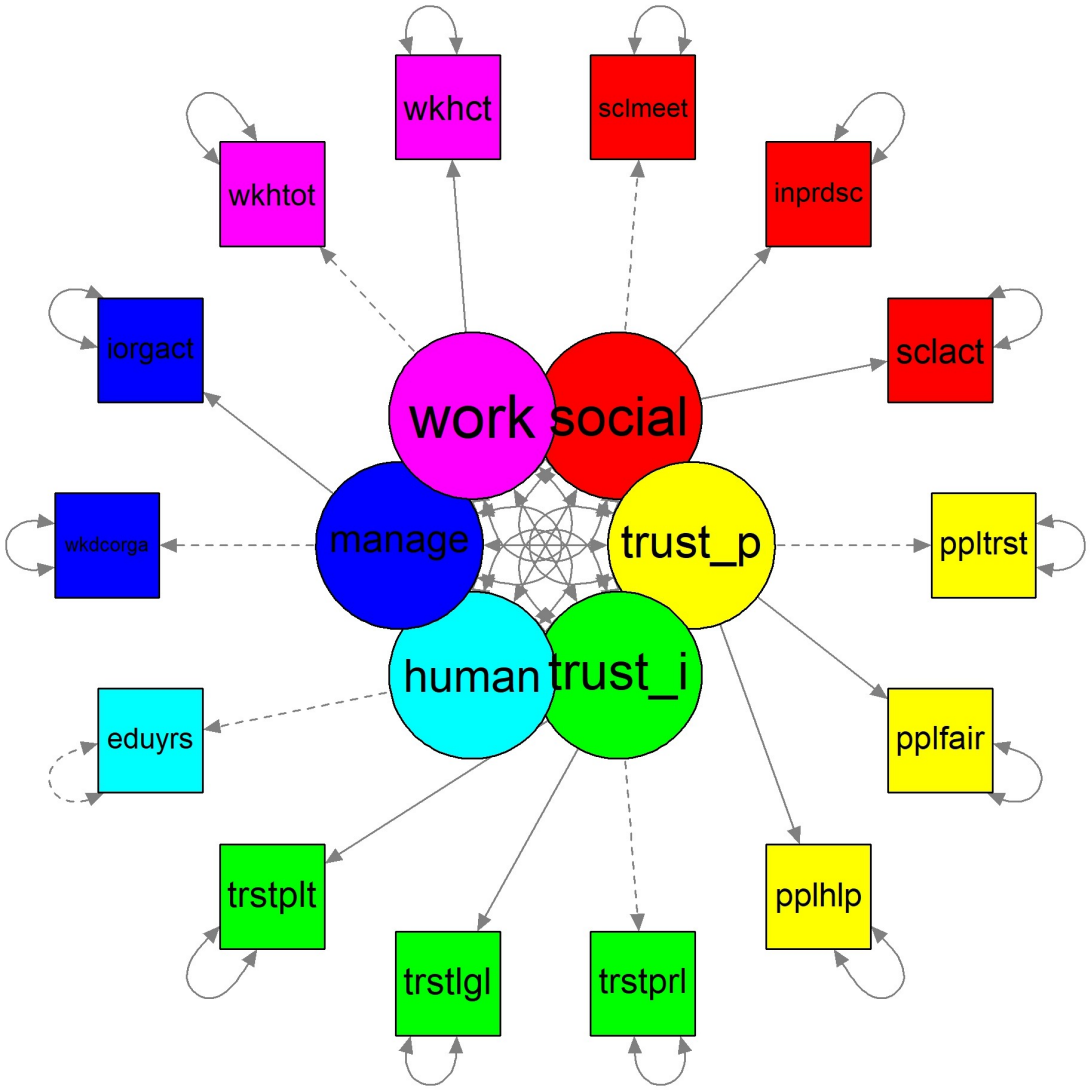
Construction of structural variables by confirmatory factor analysis. *Notes*: the inner circle represents the moderating variables. The outer circle represents raw variables included in the construction of the latent variables.

**Fig 2:**
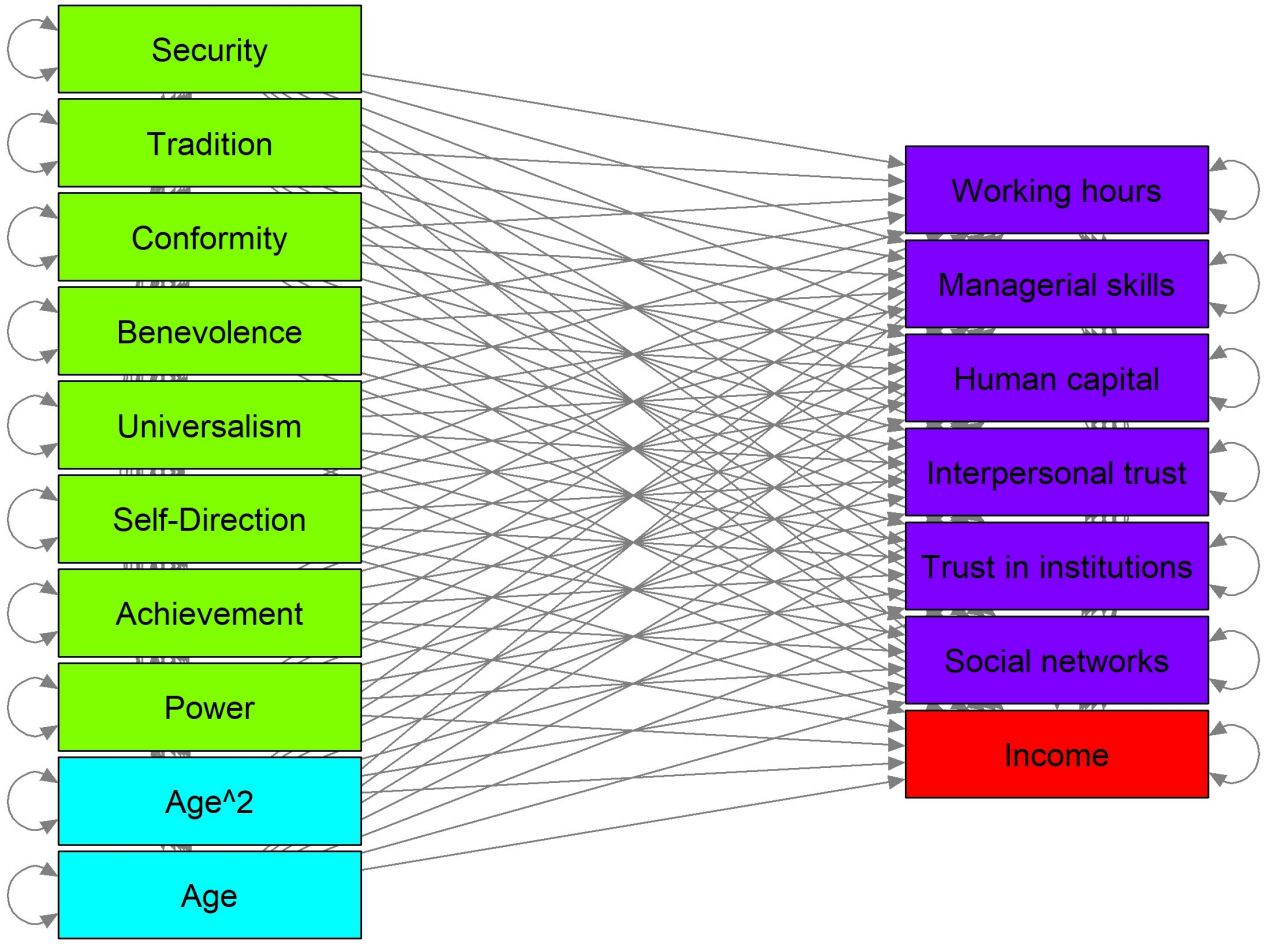
Paths in the structural equation model. *Notes*: arrows denote links in the SEM model. Income (in red) is explained by the Schwartz values (in green), age (in cyan), and the six moderating variables (in blue).

The proposed SEM model can be interpreted as a mediation model [96], with six non-causally correlated mediators (latent variables). We assume that pairwise correlations between potential mediators do not depend on the applied treatments. Thanks to this assumption indirect structural effects can be calculated in the typical way: for each mediator, the path coefficients from *V*_*i*_ on the latent variable and then from the latent variable on net income are multiplied and then summed up [96]. We allow for correlations among latent variables. For each latent factor the value of the loading for one indicator is set to one to enable identification. The construction of latent variables is presented graphically in Fig 1, whereas the paths in the SEM model are shown in Fig 2.

The structural equations, excluding constants and age-related control variables, are given by:

$$Y=\sum_{i=1}^{6}\beta_{i}X_{i\;}+\sum_{i=1}^{8}\gamma_{i}V_{i}+\varepsilon ,$$ (1)

$$X_{i}=\sum_{j=1}^{8}\alpha_{ij}V_{j}+\;\epsilon_{i},\;i\in \{1,\ldots ,6\},$$ (2)

where *Y* denotes net pay, *X*_1_ − *X*_6_ denote the latent variables, and *V*_1_ − *V*_8_ capture the eight Schwartz values. The symbols *β*_*i*_, *γ*_*i*_, *α*_*ij*_ represent the structural parameters. The variables *X*_*i*_ are non-causally correlated. The country of respondent is only indirectly considered in the model, by the manner in which the net pay and other variables were constructed (the individual income is related to the country specific mean value).

Finally, we test for the invariance of regression coefficients across European welfare state regimes, and–having rejected the null hypothesis–we proceed to carry out the analysis separately across the five European welfare state regimes.

In addition, due to the potential endogeneity of the *eduyrs* variable (individuals’ years of education; see S1 Appendix for the details), we also present the results of a modified SEM model that allows instrumental variable approach. The additional endogenous (using SEM terminology) variable representing the fathers’ level of education was introduced in the SEM base model. This variable was then used as an additional explanatory variable in the Eq (2), in which the explained variable was the *eduyrs* variable. We also allowed for the correlation between error terms of this equation and the Eq (1).

## 4. Results

In this section we first present the results of the SEM model for the full sample, and then identify the differences in results across the five European welfare state regimes.

### 4.1. Estimation of the structural equation model—Results for the full sample

The *χ*^2^ statistic for the test of equality of the observed and fitted data is statistically significant (*χ*^2^ = 1714.17, *df* = 151 and *p*-value < 0.001), suggesting bad model fit. However, *χ*^2^ test results are highly sensitive to the sample size [97] and therefore the observed significance likely results from the large sample size. Accordingly, we are able to confirm good fit of the theoretical model to empirical data based on: root mean square error of approximation (RMSEA) = 0.025 (which takes into account both model fit and degrees of freedom), comparative fit index (CFI) = 0.974, and Tucker–Lewis index (TLI) = 0.956. (Commonly used criteria for a good fit are CFI or TFI ≥.95. In practice, however, values above 0.90 for the indices are often considered to be indicative of good overall fit [91], and RMSEA ≤.06 could be considered acceptable [98]). Both CFI and TLI measure the improvement of model fit relative to the null model.

The estimated values of the key parameters in the baseline specification are presented in Table 1. The numbers are provided as percentage values, i.e. half-elasticities. The direct and total structural effects of *V*_*i*_ variables on net pay (percentage deviation from the country mean) are shown in columns 2 and 3, respectively. The effects of *V*_*i*_ variables on the latent variables are shown in the remaining columns. Statistically significant results are highlighted with asterisks. The intuitive interpretation of the results is as follows. For example, individuals who value Power (PO) more, by one category above the country mean, record on average 4.6% higher net pay, other things equal; of that, 1.8 pp. is the direct effect and the remainder is the indirect effect working through the six latent variables.

**Table 1:** Structural equation model estimation results (as percentages).

	direct	total	networks	trust inst.	trust per.	human	manage	work
PO	1.8**	4.6**	-1.2**	1.9**	-0.5**	0.3**	-0.7**	2.8**
AC	-1.1	0.9	-1**	1.4**	-0.1	0.3**	1.4**	1**
SD	-1.1	1.8**	-1.3**	0	-0.8**	0.4**	4.2**	1.2**
UN	-0.7	1.7	-1.7**	4.2**	2.9**	0.9**	-0.1	-2.4**
BE	-0.4	-1.5*	0.9**	-1.5**	-0.1	0.1	0.2	-1.8**
CO	-1	-0.9	-1.7**	2.3**	-0.5**	0	-0.1	0.5
TR	-3.3**	-4**	-1.7**	-0.4	-1.6**	-0.1**	-0.5*	0.5
SE	-0.8	-3.7**	-1.8**	-0.5*	-2.6**	-0.2**	-2.3**	-1.1**

*Notes*: PO–Power, AC–achievement, SD–self-direction, UN–universalism, BE–benevolence, CO–conformity, TR–tradition, SE–security;

* *p*<0.1,

** *p*<0.05

The respective estimates of the key parameters in the instrumental variable specification are presented in Table A.1 in the S1 Appendix. Our key results are robust to such change of specification.

The path coefficients from the latent variable to net pay are positive and significant for four out of six considered latent variables: interpersonal trust, human capital, managerial skills and hours worked (see Fig 2 and Table A.3 in the S1 Appendix). To this extent Hypothesis H1 is confirmed. However, for participation in social networks, the coefficient estimate is positive and statistically insignificant, whereas for trust in institutions the estimated value is negative and also statistically insignificant. These two latter estimates are not in line with Hypothesis H1.

With reference to Hypothesis H2 on Self-Enhancement values, we find a strong positive relationship between net pay and Power (increase in Power by one category is linked to 4,6% higher pay). Power is negatively associated with social network participation, interpersonal trust and managerial skills; it is positively associated with hours worked (in line with the literature, cf. [16]), trust in institutions and human capital. The direct and total structural effects of Power on net pay are both positive and statistically significant. However, we do not find statistically significant effects for Achievement. We only find that Achievement is positively associated with human capital, managerial skills and hours worked, but these associations are not strong enough to translate to any significant effect on net pay. Hence Hypothesis H2 is not fully confirmed.

In relation to Hypothesis H3 on Openness to Change values, we find moderately positive effects of Self-Direction. The direct effect is statistically insignificant, whereas the positive total effect follows from the fact that Self-Direction is associated with greater management skills and hours worked. Hence Hypothesis H3 is confirmed.

As regards Hypothesis H4 on Conservation values, we find a strong negative relationship between net pay and Tradition (increase in Tradition by one category is linked to 4% lower pay). Most of this negative effect (3,3 pp.) is direct, with only a minor contribution of indirect structural effects. Tradition is negatively associated with social network participation, interpersonal trust, human capital and managerial skills. A strong negative relationship is also observed between net pay and Security (increase in Security by one category is linked to 3,7% lower pay). In this case most of the negative effect (2,9 pp.) is indirect, whereas the direct effect is insignificant. Security is negatively associated with social network participation, interpersonal trust, human capital, managerial skills and hours worked. In contrast, the effects of Conformity on net pay are insignificant. Conformity is associated with greater trust in institutions but less trust in people. All in all, Hypothesis H4 is not fully confirmed. Contrary to our expectations, we obtain a direct negative effect of Tradition, whereas the total effect of Conformity is insignificant.

As for Hypothesis H5 on Self-Transcendence values, we find a moderately positive effect of Universalism. The direct effect is insignificant, whereas the positive total effect, on the verge of statistical significance, is generated through the links with selected latent variables. Universalism goes together with greater trust, both in people and institutions, greater human capital, but fewer hours worked. In turn, Benevolence goes together with greater participation in social networks and less hours worked. Owing to the latter effect, the total effect of Benevolence on net pay is moderately negative. All that means that Hypothesis H5 is not confirmed.

We also conducted a range of additional robustness checks for these results, reported in the S1 Appendix. Firstly, we estimated a linear regression model explaining net pay with age, age squared, working hours, participation in social networks, trust in institutions, interpersonal trust, managerial skills, human capital and the eight selected Schwartz values. Secondly, we estimated a linear regression model explaining net pay with the eight Schwartz values, with age as an additional control variable. The estimation results of linear regression models, together with the results of statistical testing of the data used are presented in the supplementary material. Reassuringly, the results of these estimations are comparable with the estimated total effect in the SEM model.

In the S1 Appendix we also present the results of a battery of statistical tests, indicating that the SEM model was correctly specified.

### 4.2. Results across European welfare state regimes

The above effects of personal values are intuitive and partially (though not fully) congruent with our hypotheses. But are they robust across European welfare state regimes, or are they perhaps context-dependent? To answer this question, we applied a likelihood ratio test and compared the unrestricted model (with country as a group variable) with its restricted version, where we assumed the same regression coefficients for all countries [99]. The test statistic amounts to *χ*^2^ = 3985.1 and the *p*-value is lower than 0.01. This means that there is a statistically significant difference between these two models, so that the null hypothesis that regression coefficients are the same across all countries is rejected. We proceed to the identification of differences between welfare state regimes. The results of SEM estimation across European welfare state regimes are presented in Table 2.

**Table 2:** Structural equation model estimation results across European welfare state regimes.

	Liberal	Conservative	Nordic	Mediterranean	Post-socialist
Age	0.976**	0.991**	0.854**	0.797**	0.243**
Age^2	-0.293**	-0.592**	-0.366**	-0.419**	-0.179**
Social	-0.415	-0.178	0.541	0.736	0.101
Trust inst.	-0.349**	-0.069	0.062	-0.063	-0.084**
Trust per.	0.604**	0.579**	-0.122	0.251	0.156**
Human	5.509**	3.829**	3.214**	2.616**	4.126**
Manage	0.231*	0.091*	0.314**	0.089	0.234**
Work	0.909**	0.727**	0.821**	0.723**	0.533**
PO	0.016	0.033**	-0.009	0.006	0.007
AC	-0.035	0.017*	-0.04	-0.035*	0.001
SD	-0.073**	-0.001	-0.059**	0.002	0.024**
UN	0.039	0.008	-0.027	-0.046	-0.053**
BE	-0.045	0.022	-0.078**	-0.056*	0.033**
CO	-0.022	-0.003	-0.049**	0.005	0.004
TR	-0.034	-0.036**	-0.024	0.038*	-0.067**
SE	0.01	-0.005	0.001	-0.03	-0.002

*Notes*: PO–Power, AC–achievement, SD–self-direction, UN–universalism, BE–benevolence, CO–conformity, TR–tradition, SE–security;

* *p*<0.1,

** *p*<0.05

We find that among latent variables, human capital, working hours and managerial skills have a direct positive structural effect on net pay across all the regimes. The effect of interpersonal trust is significantly positive in the liberal, conservative and post-socialist regime, while being close to zero in the Nordic and Mediterranean regime. The effect of trust in institutions is negative in the liberal and post-socialist regime and insignificant elsewhere. In turn, the effect of social network participation is universally insignificant.

Regarding the direct effects of values on net pay (presented in bottom rows of Table 2), we observe that the positive effects of Power and Achievement are significant only in the conservative regime, whereas the negative effect of Tradition is significant in the conservative and post-socialist regime. Interestingly, Self-Direction has a negative direct effect in the liberal and Nordic regime, but positive in the post-socialist regime. Benevolence has a negative direct effect in the Nordic regime, but positive in the post-socialist one. In turn, a negative and statistically significant direct effect of Conformity is observed only in the Nordic regime, while Universalism has a negative direct effect only in the post-socialist regime.

These results are robust to the potential endogeneity of the education variable, as shown in Table A.2 in the S1 Appendix.

In turn, the estimates of total (direct and indirect) effects are presented in Table 3 below.

**Table 3:** Structural equation model standardised estimation results across European welfare state regimes–total effects of variables representing Schwartz values *V*_*1*_,*…*,*V*_*8*_.

	Liberal	Conservative	Nordic	Mediterranean	Post-socialist
PO	0.052*	0.094**	0.025	-0.019	0.004
AC	-0.008	0.04**	-0.015	-0.034	0.05**
SD	-0.048*	0.027*	-0.028	0.025	0.095**
UN	0.055**	0.031**	-0.024	-0.045	-0.026*
BE	-0.026	0.006	-0.049**	-0.053*	0.025*
CO	-0.031	-0.006	-0.032	-0.005	0.003
TR	-0.061**	-0.045**	-0.022	0.032	-0.143**
SE	-0.049**	-0.045**	-0.026	-0.053**	-0.029**

*Notes*: PO–Power, AC–achievement, SD–self-direction, UN–universalism, BE–benevolence, CO–conformity, TR–tradition, SE–security;

* *p*<0.1,

** *p*<0.05

We find the following statistically significant results. Power, which is strongly positively associated with economic performance (net pay) in the full sample, is actually relevant primarily in the conservative and liberal regime. In turn, Tradition and Security, negatively associated with net pay in the full sample, remain relevant primarily in the post-socialist, liberal and conservative regime. Universalism is positively related to economic performance in the liberal and conservative regime, but negatively in the post-socialist regime. Finally, Self-Direction is positively related to net pay in the post-socialist and conservative regime, while it is negatively related in the liberal regime.

This means that none of the results are fully robust result across all five European welfare state regimes. The results are generally context-sensitive. Furthermore, most total effects of values in the Nordic and Mediterranean welfare state regime are statistically insignificant, suggesting that the link between values and economic performance is weak there (or there is relatively more noise in the reporting of Schwartz values or net incomes there).

According to Esping-Andersen the liberal welfare state regime promotes Power, Achievement and Self-Direction, the conservative one promotes Power, Security and Tradition, whereas the Nordic regime promotes Universalism [18]. For this reason we hypothesised that Power should be most effective in raising net pay in the liberal and conservative welfare state regime because it is a guiding principle of the whole stratification system there. Therefore action guided by this value might be socially more approved and justified. But our results are mixed: we find that Power indeed leads to higher net pay in the liberal and conservative welfare state regime; however, Self-Direction in the liberal regime and Tradition and Security in the conservative (and liberal) regime are negatively related to income. Achievement in the liberal welfare state regime is statistically insignificant. Furthermore, our analysis reveals that the other Schwartz values may lead to higher net pay in these regimes: Universalism in the liberal and conservative welfare state regimes, Achievement and Self-Direction in the conservative welfare state regime. A potential reason for this phenomenon (positive correlation of the Schwartz values opposing the ones promoted by the given welfare state regime) may be a (monetary) premium for nonconformity. Furthermore, Universalism has no statistically significant effect on net pay in the Nordic regime; only Benevolence is significantly negatively associated with net pay there. In contrast, Benevolence, the Schwartz value promoted by in the Mediterranean welfare state, is negatively associated with net pay there. All in all, Hypothesis H6 is firmly rejected.

Similarly as for the full sample, we also conducted additional robustness checks for our results across European welfare state regimes. Firstly, we estimated a linear regression model explaining net pay with the age, age squared, working hours, participation in social networks, trust in institutions, interpersonal trust, managerial skills, human capital and the eights selected Schwartz values, for each regime separately. Secondly, we estimated a linear regression model explaining net pay with the eight Schwartz values, with age as a control variable, for each regime separately. The results of the robustness checks are comparable with the estimated total effect in the SEM model.

## 5. Discussion

Results from our SEM model, which can be interpreted as a mediation model with six non-causally correlated mediators (latent variables: social network participation, interpersonal trust, trust in institutions, human capital, managerial skills and working hours), imply that the ordering of values in terms of the magnitude of their relation to net pay is different in the case of direct and total effects, and that indirect effects are often sizeable.

Specifically, greatest differences between direct and total effects are observed for Self-Direction and Security. None of these two variables exhibits a significant direct effect on economic performance. However, the indirect structural effects of both variables are sizeable. Self-Direction is positively linked to management skills, hours worked and human capital (with a small negative effect on interpersonal trust), contributing to a significant positive total effect of Self-Direction. In turn, Security is negatively linked to management skills, hours worked and interpersonal trust, contributing to a significant negative total effect of Security.

Our results confirm our initial hypothesis that individuals whose values are focused around Self-Enhancement values such as Power are particularly motivated to have higher income, since they value extrinsic rewards from work [16, 78, 86]: as expected, we find both a direct positive effect of this value and an indirect structural effect through hours worked. For individuals whose values are focused around Achievement–the second Self-Enhancement value–we do not find a significant positive effect, though, despite the fact that an indirect effect on hours worked (as well as managerial skills) is clearly there.

Our results are also congruent with the assertion that people who have an aggressive entrepreneurial personality prefer autonomy, risk-taking and changes to the *status quo* [82, 83]. They praise the value of Self-Direction over Security. An individual that is motivated by Self-direction is free, creative, curious and independently chooses one’s own goals. In line with this, we find that people driven by Self-Direction work longer hours and have better managerial skills; for Security, the opposite holds. Probably, in the case of individuals motivated by Self-Direction, their life choices reflect their beliefs, values and desires to a greater extent than for those motivated by Security [78, 86, 100]. These conditions indirectly lead to higher net pay.

Our results also add a caveat to the initial hypothesis that all Conservatism values (Tradition, Security, Conformity) have similar consequences; in particular, Tradition exhibits similar effects as Security. We find that the overall effect for these two values is indeed similar, i.e. negative and statistically significant, but for Tradition this effect is due to a direct effect, and for Security–an indirect one.

Our Europe-wide results are summarized schematically in Table 4 below.

**Table 4:** Summary of hypotheses H2-H5 and their verification.

	direct	networks	trust inst.	trust per.	Human	manage	work
Hypotheses H2-H5							
Self-Enhancement	+*						+*
Openness to Change				+	+*	+*	+*
Conservatism				-*	-*	-*	-*
Self-Transcendence							
SEM Results							
Self-Enhancement	+	-^a^	+^a^	-	+	?	+
Openness to Change		-^a^		-	+	+	+
Conservatism	-	-^a^	? ^a^	-	-	-	-
Self-Transcendence		-^a^	? ^a^	+	+		-

*Notes*: Self-Enhancement: Power, Achievement; OC–Openness to Change: Self-Direction; Conservatism: Tradition, Security, Conformity; Self-Transcendence: Universalism, Benevolence.

“*” indicates confirmed hypotheses. “?” denotes ambiguous results (different signs for different Schwartz values in a given group).

^a^Recall that, contrary to Hypothesis H1, social network participation and trust in institutions have turned out to be statistically insignificant predictors of incomes.

At the level of welfare state regimes our analysis provides mixed results, suggesting that while the identified effects are context-dependent, the resulting patterns of dependence are not always quite intuitive or consistent with established theory. Note, however, that our analysis focuses only on the effects in a cross-section of individuals living within a certain welfare state regime, and owing to the construction of our variables we cannot draw any conclusions at the societal level.

Schwartz claimed that societies which emphasize Hierarchy and Mastery (respectively, Power and Achievement on an individual level) enhance individuals within a society to think of work as central to their lives [4, 16, 34]. As a consequence, this may lead to greater devotion to work and in effect to higher net pay. Similar relation stems from Lin’s theory of social action [101]. If the system is an occupational or economic class pyramid, it generates motivation for gaining more authority and for social climbing [86]. In ESS data, however, Power and Achievement values are most common (statistically significantly above mean) in the liberal and Nordic welfare state regimes. This results seems counterintuitive given the strong decommodification in the Nordic regime. In turn, Achievement values are least common (significantly below mean) in the conservative and Mediterranean one. In our study we find that–cross-cutting these rankings–Power is associated with higher net pay in the liberal and conservative regime, whereas Achievement is associated with higher net pay in the conservative and post-socialist regime.

Schwartz’s assertion that extrinsic goals (e.g., pay) are most important for people in cultures that emphasise Conservatism (Tradition and Security at the individual level), and least important in cultures that promote Intellectual Autonomy (Self-Direction at the individual level) [16], is not fully confirmed either. Across welfare state regimes, Self-Direction is particularly high and Security is particularly low in the post-socialist regime (significantly above and below mean, respectively), whereas Tradition is high in the conservative regime (significantly above mean). This result contradicts initial expectations as high focus on Self-Direction was expected rather in the liberal regime than in the post-socialist regime where decommodification is weak. However, we find that within the post-socialist and conservative regime–like in the full sample–Self-Direction is associated with higher pay, whereas Security and Tradition are associated with lower pay. Self-Direction, as opposed to Security, governs people’s actions in such a way as to create contexts that lead to higher net pay, even though higher net pay may not be their direct goal. As Kadushin puts it, referring to the effectiveness drive: “actors need not be consciously aware of these structurally similar others to behave as if they were trying to keep up with them” [86].

In sum, some of our results for European welfare state regimes may be puzzling, in particular when compared to the earlier discussions by Schwartz [16] and Kadushin [86]. Nevertheless, the latter author already anticipated the possibility of such outcomes: “By and large, we can assume that persons and collectivities that are congruent with the dominant culture/personality mode will be more valued. Going ‘outside the box’ however (…) can be dramatically effective if there is also a cultural prescription that allows for such relatively unusual behavior” ([86], p. 66). Kadushin’s claim relates to our results in two ways. Firstly, Hypothesis H6 stresses monetary pay-offs from the congruency between personal values and the dominant culture, whereas in reality the pay-offs might be also non-monetary, in form of, e.g., respect or sense of belonging. Secondly, Hypothesis H6 may understate the importance of scenarios of “going outside the box” (i.e., behaviours that are rare in a given community but are potentially rewarded with a premium) in some of the regimes, which disturb the clear pattern of relationship between personal values and their pay-offs.

## 6. Conclusion

What underlies the variation in individuals’ economic performance, as measured by their incomes? To which extent can this variation be explained by the values that people hold? In this paper we have studied the direct and indirect links between people’s values and their economic performance. We considered indirect effects through moderating variables such as social network participation, interpersonal trust, trust in institutions, human capital, managerial skills and hours worked. These links were studied using structural equation modelling (SEM) methodology applied to European Social Survey data from 28 European countries in 2018. Schwartz classification of values was used, distinguishing between Self-Enhancement (Power, Achievement), Openness to Change (Self-Direction), Conservation (Tradition, Security, Conformity) and Self-Transcendence (Universalism, Benevolence) values. It was found that Power has the strongest positive direct effect on economic performance, further strengthened by a positive indirect structural effect through hours worked. Self-Direction is indirectly positively linked to economic performance through higher managerial skills and hours worked. Tradition has a strong negative direct effect on economic performance. Security is indirectly negatively linked with economic performance, owing to its negative effects on interpersonal trust, management skills and hours worked. Finally, some of the identified effects are context-dependent and vary across European welfare state regimes.

Our findings lend themselves to several policy implications, particularly in relation to the policies on immigration, demographics, the labor market, and work-life balance. Specifically, we expect that immigrant acculturation will be particularly difficult if the immigrants’ values (e.g., Tradition) not only do not coincide with the values promoted in the host country (e.g., Power), but also are conducive to lower incomes in the host country. Such mismatch of values then adds to the known variety of other reasons why immigrants’ incomes tend to be lower than those of the natives, ranging from language barriers and skill mismatches to labor market discrimination. Furthermore, if the immigrants decide to modify their values to better fit in the host society, that requires psychological effort associated with stress. In turn, the link of our results to demographic policy consists in highlighting the differential difficulty in pursuing such policy, depending on the dominant values in a given society and their link to economic performance. Policies aimed at increasing the fertility rate will be particularly difficult to implement in countries where Power and Achievement are both important and associated with higher incomes, as it is the case in the conservative welfare state regime. In the labor market the identified links between values and economic performance suggest that, other things equal, liberalization policies will have relatively more pronounced effects in the conservative and post-socialist welfare state regimes, where Achievement and Self-Direction are linked with higher incomes. Our results suggest that also the policy of work-life balance promotion should be tailored to the different welfare state regimes. For example, welfare state regimes that promote Power, and where Power is actually correlated with better economic performance, will be particularly reluctant to introduce work-life balance policies (e.g., introducing flexible, family-friendly working arrangements).

The are several limitations of our study. Firstly, due to the cross-sectional character of the dataset, we were unable to identify causal relations among the variables of interest. Secondly, the study relies on self-reported values. (We consider this a limitation, even though the ESS is widely acknowledged as a high-quality source of survey data.) Thirdly, we only use data from European countries. Despite notable differences, these countries may be viewed as culturally quite homogeneous when compared to countries from other continents. Fourth, the selection of variables for our SEM model was to a certain extent arbitrary, conditioned by the availability of data, and so were some decisions on the statistical procedure, e.g., leaving outlier observations in the sample. To minimise the impact of arbitrariness on our results, we did our best to base the construction of the model on the theoretical literature as well as conducted additional robustness checks.

The research performed in this study can be extended in several directions. First, one could assess robustness of our results using other datasets, e.g., covering other countries or capturing the temporal variation of the considered variables. Second, one could consider other outcomes than earnings, e.g., it is also interesting how values may affect individuals’ subjective well-being. Third, one could conduct experiments aimed at better gauging how values causally affect outcomes in a controlled setting.

## Supporting information

S1 Appendix Contains all the supplementary files for this submission. (PDF)

S1 Checklist Human participants research checklist. (DOCX)
