# Supplementary material for: Values and economic performance across European welfare state regimes: Direct and indirect effects through social capital, human capital and managerial skills
Source: PLoS One. 2024 Feb 23;19(2):e0298667. doi: 10.1371/journal.pone.0298667 (PMC10889612; doi:10.1371/journal.pone.0298667)
Supplement: S1 Appendix — (PDF) [file pone.0298667.s001.pdf]

Supporting information

A.1. Instrumental variable structural equation model estimation results

Table A.1: Instrumental variable structural equation model estimation results (as percentages), controlling for the endogeneity of education.

	direct	total	network	trust inst.	trust pers.	human	manage	work
PO	0.5	4.6**	-1.2**	1.9**	-0.5**	0.3**	-0.6**	2.8**
AC	-2.2**	0.9	-1**	1.4**	-0.1	0.3**	1.4**	1**
SD	-2.8**	1.9**	-1.3**	0	-0.8**	0.4**	4.2**	1.3**
UN	-3.9**	1.8*	-1.7**	4.3**	3**	0.9**	0	-2.3**
BE	-0.4	-1.5*	0.9**	-1.5**	-0.1	0.1	0.2	-1.8**
CO	-1.4**	-0.9	-1.7**	2.3**	-0.5**	0	-0.1	0.5
TR	-3.5**	-4**	-1.7**	-0.4	-1.6**	-0.1**	-0.5*	0.5
SE	-0.9	-3.7**	-1.8**	-0.4*	-2.6**	-0.2**	-2.3**	-1.1**

Notes: PO – Power, AC – achievement, SD – self-direction, UN – universalism, BE – benevolence, CO – conformity, TR – tradition, SE – security; * $p<0.1$, ** $p<0.05$

Table A.2: Instrumental variable structural equation model estimation results across European welfare state regimes.

	Liberal	Conservative	Nordic	Mediterranean	Post-socialist
age	0.992**	1.106**	0.923**	0.816**	0.325**
age^2	-0.237	-0.469**	0.052	-0.409**	-0.089**
network	-0.487	-0.48**	0.163	0.646	-0.215
trust inst.	-0.378**	-0.168**	-0.229	-0.063	-0.062*
trust pers.	0.57**	0.514**	-0.322	0.235	0.018
human	7.151**	8.219**	13.866**	3.059**	9.801**
manage	0.216*	0.003	0.233*	0.08	0.112**
work	0.903**	0.706**	0.769**	0.724**	0.598**
PO	0.01	0.013	-0.042	0.003	0.001
AC	-0.042	0.009	-0.088**	-0.037*	-0.025**
SD	-0.077**	-0.018	-0.102**	-0.001	-0.013
UN	0.026	-0.03	-0.101**	-0.052	-0.085**
BE	-0.05	0.022	-0.092**	-0.055*	0.03**
CO	-0.022	-0.004	-0.064**	0.004	-0.01
TR	-0.034	-0.041**	-0.022	0.038*	-0.072**
SE	0.008	0	-0.004	-0.031	-0.009

Notes: PO – Power, AC – achievement, SD – self-direction, UN – universalism, BE – benevolence, CO – conformity, TR – tradition, SE – security; * $p<0.1$, ** $p<0.05$

A.2. Raw structural equation model estimates

Table A.3: Structural equation model estimation results (factor loadings and regression coefficients)

lhs	op	rhs	Est	se	z	*p*-value
income	=~	netinum	1	0		
social	=~	sclmeet	1	0		
social	=~	inprdsc	0.9635	0.0315	30.5405	0
social	=~	sclact	0.6	0.0186	32.294	0
trust_p	=~	ppltrst	1	0		
trust_p	=~	pplfair	0.8301	0.0152	54.5627	0
trust_p	=~	pplhlp	0.6764	0.014	48.2433	0
trust_i	=~	trstprl	1	0		
trust_i	=~	trstlgl	0.8136	0.0105	77.2452	0
trust_i	=~	trstplt	0.852	0.0094	90.7465	0
human	=~	eduyrs	1	0		
manage	=~	wkdcorga	1	0		
manage	=~	iorgact	1.0618	0.027	39.3206	0
work	=~	wkhtot	1	0		
work	=~	wkhct	0.7099	0.0178	39.8946	0
income	~	age	0.8794	0.0429	20.506	0
income	~	age^2	-0.4652	0.0306	-15.2125	0
income	~	social	-0.0577	0.1144	-0.5045	0.6139
income	~	trust_i	-0.0235	0.031	-0.7573	0.4489
income	~	trust_p	0.3639	0.055	6.6193	0
income	~	human	3.7315	0.1529	24.4077	0
income	~	manage	0.1459	0.0248	5.8732	0
income	~	work	0.772	0.0226	34.1375	0
income	~	PO	0.0175	0.0068	2.5649	0.0103
income	~	AC	-0.0106	0.0066	-1.6	0.1096
income	~	SD	-0.0114	0.0074	-1.5371	0.1243
income	~	UN	-0.0067	0.0099	-0.6769	0.4985
income	~	BE	-0.0036	0.0087	-0.4159	0.6775
income	~	CO	-0.01	0.0057	-1.7668	0.0773
income	~	TR	-0.0331	0.0067	-4.938	0
income	~	SE	-0.0084	0.0064	-1.3093	0.1904
social	~	age	-0.0612	0.0067	-9.0719	0
social	~	age^2	0.0128	0.0046	2.7978	0.0051
social	~	PO	-0.0122	0.0011	-10.6723	0
social	~	AC	-0.01	0.001	-9.5511	0
social	~	SD	-0.0125	0.0013	-9.8001	0
social	~	UN	-0.0167	0.0015	-10.8468	0
social	~	BE	0.0094	0.0015	6.3324	0
social	~	CO	-0.0175	0.001	-17.9347	0

social	~	TR	-0.0169	0.0011	-15.9389	0
social	~	SE	-0.0176	0.0011	-16.6391	0
trust_i	~	age	-0.0223	0.0144	-1.5411	0.1233
trust_i	~	age^2	0.0146	0.0096	1.5127	0.1304
trust_i	~	PO	0.0189	0.0025	7.5943	0
trust_i	~	AC	0.0136	0.0023	5.9212	0
trust_i	~	SD	-0.0004	0.0028	-0.1462	0.8838
trust_i	~	UN	0.0424	0.0034	12.438	0
trust_i	~	BE	-0.015	0.0032	-4.6089	0
trust_i	~	CO	0.023	0.0021	10.8441	0
trust_i	~	TR	-0.0037	0.0023	-1.5779	0.1146
trust_i	~	SE	-0.0045	0.0023	-1.9442	0.0519
trust_p	~	age	0.0986	0.0124	7.9753	0
trust_p	~	age^2	-0.0119	0.0083	-1.4321	0.1521
trust_p	~	PO	-0.0051	0.0022	-2.3363	0.0195
trust_p	~	AC	-0.0014	0.002	-0.6913	0.4894
trust_p	~	SD	-0.0083	0.0024	-3.4646	0.0005
trust_p	~	UN	0.0295	0.0028	10.3465	0
trust_p	~	BE	-0.0008	0.0028	-0.2866	0.7744
trust_p	~	CO	-0.0052	0.0018	-2.8578	0.0043
trust_p	~	TR	-0.0161	0.002	-8.1021	0
trust_p	~	SE	-0.0264	0.002	-13.4352	0
human	~	age	-0.0209	0.0022	-9.4494	0
human	~	age^2	-0.032	0.0016	-19.7767	0
human	~	PO	0.0025	0.0004	7.0197	0
human	~	AC	0.0027	0.0003	8.3934	0
human	~	SD	0.0043	0.0004	10.7451	0
human	~	UN	0.0086	0.0005	18.2937	0
human	~	BE	0.0007	0.0005	1.5288	0.1263
human	~	CO	-0.0003	0.0003	-0.8952	0.3707
human	~	TR	-0.0013	0.0003	-3.9886	0.0001
human	~	SE	-0.0022	0.0003	-6.7554	0
manage	~	age	0.2796	0.0192	14.5545	0
manage	~	age^2	-0.1739	0.0139	-12.5396	0
manage	~	PO	-0.0065	0.0031	-2.0928	0.0364
manage	~	AC	0.0136	0.0028	4.7929	0
manage	~	SD	0.0415	0.0035	11.994	0
manage	~	UN	-0.0007	0.0041	-0.1823	0.8553
manage	~	BE	0.002	0.004	0.502	0.6157
manage	~	CO	-0.0014	0.0026	-0.5473	0.5842
manage	~	TR	-0.0055	0.0029	-1.9002	0.0574
manage	~	SE	-0.0227	0.0028	-8.1652	0
work	~	age	0.4366	0.0306	14.253	0
work	~	age^2	-0.4773	0.0258	-18.5341	0

work	~	PO	0.0281	0.0045	6.2325	0
work	~	AC	0.0098	0.0043	2.2716	0.0231
work	~	SD	0.0125	0.0048	2.6141	0.0089
work	~	UN	-0.0238	0.0058	-4.1146	0
work	~	BE	-0.0184	0.0055	-3.3431	0.0008
work	~	CO	0.0047	0.0038	1.257	0.2087
work	~	TR	0.0052	0.0043	1.2175	0.2234
work	~	SE	-0.0108	0.004	-2.7226	0.0065

Notes: PO – Power, AC – achievement, SD – self-direction, UN – universalism, BE – benevolence, CO – conformity, TR – tradition, SE – security.

A.3. Additional robustness checks and statistical tests

We verified the assumptions and conducted the robustness checks based on the same dataset, which we used for structural equation modelling (n = 16435). For the sake of this robustness check we additionally constructed the following variables: participation in social networks (as the arithmetic average of original ESS variables: *sclmeet*, *inprdsc* and *sclact*), interpersonal trust (average of *ppltrst*, *pplfair* and *pplhlp*), trust in institutions (average of *trstprl*, *trstlgl* and *trstplt*) and managerial skills (average of *wkdcorga* and *iorgact*), as equivalents of the latent variables created in structural equation models.

We are not aware of past studies identifying valid and strong instruments for individuals' values, particularly that values are known to be persistent and determined largely in people's youth. However, earlier literature (e.g., [102-103]) discusses the problem of endogeneity of years of education in the wage regression. The problem may be caused by the omitted variable "ability". In such a case two approaches are recommended: using a proxy variable or instrumental variables approach. As no proxy variable was available in the ESS dataset, we followed the latter approach and selected *edulvlfb* (father's highest level of education) and *edulvlmb* (mother's highest level of education) as potential instrumental variables based on the literature and data availability. Missing values for the instrumental variables – respectively 9.49% and 6.83% of the cases – were imputed using the respective country averages.

As a side note, we also initially stipulated the endogeneity of two further variables: *manage* and *wkhtot* (toal number of working hours) due to omitted variables: resourcefulness in the former and diligence in the former case. However, empirical testing using instrumental variables: *actrolga* (able to take active role in political group) and *sofrwrk* (society fair when hard-working people earn more than others) provided no evidence for endogeneity. There is no empirical evidence in the literature nor theoretical considerations that may suggest the endogeneity of the remaining variables.

We estimated two regression models. In the first model net income was regressed on the following explanatory variables: age, age squared, working hours, participation in social networks, trust in institutions, interpersonal trust, managerial skills, human capital and the eights selected Schwartz values. In the second reduced model only age, age squared and eight selected Schwartz values were used. To avoid structural multi-collinearity the age variable was demeaned before squaring.

In the first stage we tested for residual normality, homogeneity and the lack of regressor multicollinearity.

Rigorous testing by means of Kolmogorov-Smirnov test provides the empirical evidence for non-normality of the residuals terms in the regression equation. However, the visual inspection of the residuals histogram (see Figure A.1) and the QQ plot makes it possible

to assume an approximately normal distribution of the residuals with additional outliers (an alternative option would be to assume a leptokurtic distribution). Therefore, we also test for the presence of outliers in the data by the means of Bonferroni outlier test [104-105], assuming a *p*-value of 5% for the outliers detection.

Figure A.1 Histogram of the residuals

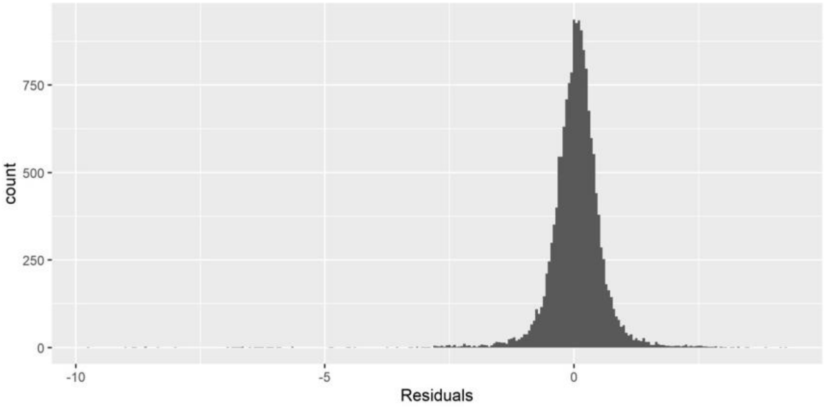

The results of the tests are presented in Table A.4.

Table A.4: The results of the outliers detection, multi-collinearity and heteroskedasticity test

Regime	N	N Outl.	% Outl.	VIF	BP *p*-value
Liberal	1382	12	0.87	1.65	0.2277
Conservative	4716	37	0.78	1.61	0
Nordic	2430	22	0.91	1.7	0
Mediterranean	1569	19	1.21	1.62	0.6077
Post-socialist	6338	34	0.54	1.57	0
All	16435	128	0.78	1.51	0

Note: Results of the statistical testing. The following columns present: the welfare state regime, total number of outliers, percentage of outliers, maximal value of Variance Inflation Factors and the *p*-values of the Breusch-Pagan test for residual heteroskedasticity [106].

The results provide no evidence of multi-collinearity. However, heteroskedasticity of residuals is present in all but Liberal and Mediterranean regimes. For these cases, we follow White's approach [107] and use the heteroskedasticity-consistent covariance matrix for the calculation of the estimation errors further on. As the percentage of detected outliers is limited we decided to conservatively leave the outliers in the sample in order to avoid over-estimating the fit of the model to the data.

In the second stage, we tested for the endogeneity of *eduyrs* (years of schooling) using both regression-based and original Hausman's tests [103]. The first test allows for residual heteroskedasticity; the results of the second test are only shown for informational purposes. The results of the Sargan test [108] indicate that only one of the two instrumental variables should be chosen. We selected *edulvlfb* (father's education) based on the statistical properties of the variable (higher value of the correlation coefficient with the individual's human capital level). The results are presented in Table A.5.

Table A.5: Endogeneity test results

Regime	*p*-value of regression based Hausman test	*p*-value of original Hausman test
Liberal	0.987	0.987
Conservative	0.083	0.009*
Nordic	0.014*	0.129
Mediterranean	0.799	0.799
Post-socialist	0.014*	. <0.001*
All	0.037*	0.001*

The results of the tests are somewhat ambiguous. In case of the following welfare state regimes: Liberal, Conservative and Mediterranean endogeneity is not detected, but it is detected in case of the other regimes: Nordic and Post-socialist, and additionally for the whole dataset. Therefore, in the following part we present the estimation results of both: ordinary least squared and instrumental variables regressions for all the regions in order to preserve comparability.

The OLS regression results for the whole dataset are shown in Table A.6, whereas the IV regression results are shown in Table A.7.

Table A.6: Ordinary least squares regression results for the whole dataset

	Estimate	Std. Error	*t* value	Pr(>|t|)
(Intercept)	-0.101	0.011	-9.016	0
agea_demean	0.977	0.075	13.041	0
agea_2_demean	-5.438	0.482	-11.288	0
Wkhtot	0.605	0.029	21.12	0
Eduyrs	3.819	0.281	13.571	0
Networks	-0.007	0.107	-0.061	0.952
trust_people	0.271	0.059	4.608	0
trust_institution	0.008	0.044	0.174	0.862
manage	0.129	0.034	3.823	0
PO	0.024	0.011	2.13	0.033
AC	-0.011	0.011	-0.999	0.318
SD	-0.013	0.014	-0.946	0.344
UN	-0.008	0.018	-0.452	0.651
BE	-0.011	0.017	-0.655	0.513
CO	-0.009	0.009	-1.007	0.314
TR	-0.035	0.014	-2.511	0.012
SE	-0.009	0.01	-0.871	0.384

Notes: PO – Power, AC – achievement, SD – self-direction, UN – universalism, BE – benevolence, CO – conformity, TR – tradition, SE – security.

Table A.7: Instrumental variable regression results for the whole dataset

	Estimate	Std. Error	*t* value	Pr(>|t|)
(Intercept)	-0.117	0.014	-8.577	0
agea_demean	1.053	0.086	12.288	0
agea_2_demean	-4.602	0.622	-7.399	0
wkhtot	0.595	0.029	20.848	0
eduyrs	6.794	1.388	4.896	0
network	-0.144	0.127	-1.129	0.259
trust_people	0.206	0.066	3.115	0.002
trust_institution	-0.038	0.05	-0.747	0.455
manage	0.082	0.04	2.048	0.041
PO	0.015	0.012	1.234	0.217
AC	-0.019	0.012	-1.622	0.105
SD	-0.025	0.015	-1.711	0.087
UN	-0.032	0.021	-1.556	0.12
BE	-0.013	0.017	-0.742	0.458
CO	-0.01	0.009	-1.049	0.294
TR	-0.034	0.014	-2.49	0.013
SE	-0.008	0.011	-0.708	0.479

Notes: PO – Power, AC – achievement, SD – self-direction, UN – universalism, BE – benevolence, CO – conformity, TR – tradition, SE – security.

The OLS regression results for different welfare state regimes are shown in Table A.8.

Table A.8: Ordinary least squares regression results for different welfare state regimes

	**netinum**	**netinum**	**netinum**	**netinum**	**netinum**
Predictors	*Estimates*	*Estimates*	*Estimates*	*Estimates*	*Estimates*
(Intercept)	-0.335 ***	-0.032 **	-0.125 ***	-0.140 ***	-0.067 ***
agea demean	1.107 ***	1.080 ***	0.909 ***	0.907 ***	0.281 **
agea 2 demean	-4.545 ***	-6.359 ***	-3.925 *	-5.037 ***	-1.983 *
wkhtot	0.643 ***	0.647 ***	0.806 ***	0.454 ***	0.269 ***
eduyrs	5.792 ***	3.990 ***	3.282 ***	2.595 ***	4.118 ***
network	-0.350	-0.062	0.349	0.301	0.053
trust people	0.373 *	0.367 ***	-0.093	0.230 *	0.133 .
trust institution	-0.287 *	0.026	0.065	-0.043	-0.064 *
manage	0.228 *	0.061	0.208 *	0.097 .	0.218 ***
PO	0.017	0.040 **	-0.009	0.001	0.011

AC	-0.038	0.015	-0.037	-0.037 .	0.003
SD	-0.090 *	-0.001	-0.062	0.004	0.024
UN	0.039	0.011	-0.039	-0.067 .	-0.051 *
BE	-0.061	0.018	-0.083 *	-0.053 .	0.023
CO	-0.024	-0.001	-0.055 .	-0.001	0.001
TR	-0.045	-0.038 *	-0.026	0.039	-0.071 ***
SE	-0.008	-0.004	0.012	-0.035	-0.003
Observations	1382	4716	2430	1569	6338
R^2 / R^2 adjusted	0.254 / 0.245	0.441 / 0.439	0.274 / 0.269	0.176 / 0.167	0.179 / 0.177

*. p<0.1 * p<0.05 ** p<0.01 *** p<0.001*

Notes: PO – Power, AC – achievement, SD – self-direction, UN – universalism, BE – benevolence, CO – conformity, TR – tradition, SE – security.

The IV regression results for different welfare state regimes are shown in Table A.9.

Table A.9: Instrumental variables regression results for different welfare state regimes

	**netinum**	**netinum**	**netinum**	**netinum**	**netinum**
Predictors	*Estimates*	*Estimates*	*Estimates*	*Estimates*	*Estimates*
(Intercept)	-0.292 ***	-0.049 **	-0.218 **	-0.143 ***	-0.073 ***
agea demean	0.751 ***	1.168 ***	0.943 ***	0.929 ***	0.342 ***
agea 2 demean	-4.379 ***	-5.442 ***	-0.479	-4.911 ***	-1.370 .
wkhtot	0.824 ***	0.629 ***	0.755 ***	0.453 ***	0.282 ***
eduyrs	4.257 **	7.267 ***	12.396	3.064	8.449 ***
network	0.008	-0.207 .	0.191	0.262	-0.097
trust people	0.227 **	0.305 **	-0.264	0.219*	0.034
trust institution	-0.013	-0.051	-0.188	-0.047	-0.062
manage	0.350 ***	0.018	0.158 .	0.087	0.143 **
PO	0.025 .	0.025 .	-0.035	0.001	0.009

AC	-0.023 .	0.009	-0.078 .	-0.038 .	-0.016
SD	-0.054 ***	-0.015	-0.094 *	0.002	-0.005
UN	-0.003	-0.017	-0.103 .	-0.072 .	-0.075 **
BE	-0.029 .	0.017	-0.094 *	-0.053 .	0.020
CO	-0.010	-0.001	-0.066 *	-0.001	-0.008
TR	-0.034 **	-0.040 *	-0.020	0.040 .	-0.070 ***
SE	-0.016	0.001	0.010	-0.035	-0.006
Observations	1382	4716	2430	1569	6338
R^2 / R^2 adjusted	0.234 / 0.225	0.421 / 0.419	0.197 / 0.192	0.175 / 0.166	0.124 / 0.122

*. p<0.1 * p<0.05 ** p<0.01 *** p<0.001*

Notes: PO – Power, AC – achievement, SD – self-direction, UN – universalism, BE – benevolence, CO – conformity, TR – tradition, SE – security.

The results of the outliers detection, multi-collinearity and heteroskedasticity test for the IV regression are shown in Table A.10.

Table A.10: The results of the outliers detection, multi-collinearity and heteroskedasticity test for the instrumental variables regression.

Regime	N	N Outl.	% Outl.	VIF	BP *p*-value
Liberal	1382	10	0.72	1.59	0.2067
Conservative	4716	28	0.59	1.51	0
Nordic	2430	23	0.95	1.63	0
Mediterranean	1569	20	1.27	1.57	0.0002
Post-socialist	6338	41	0.65	1.55	0
All	16435	126	0.77	1.49	0

Note: Results of the statistical testing. The following columns present: the welfare state regime, total number of outliers, percentage of outliers, maximal value of Variance Inflation Factors and the *p*-values of the Breusch-Pagan test for residual heteroskedasticity [106].

These results provide no evidence for multi-collinearity. Heteroskedasticity is present in all but the Liberal regime's dataset.

For completeness, we also estimated the reduced model. The results correspond to the estimated total effect in the structural equations model and are presented in Table A.11 (for the whole sample) and Table A.12 (across European welfare state regimes).

Table A.11: Ordinary least squares regression results for the whole dataset

	Estimate	Std. Error	*t* value	Pr(>|t|)
(Intercept)	-0.063	0.013	-4.958	0
agea_demean	1.219	0.082	14.931	0
agea_2_demean	-9.839	0.624	-15.759	0
PO	0.046	0.013	3.681	0
AC	0.009	0.012	0.722	0.47
SD	0.018	0.015	1.197	0.231
UN	0.017	0.02	0.873	0.383
BE	-0.015	0.019	-0.826	0.409
CO	-0.009	0.011	-0.837	0.402
TR	-0.04	0.015	-2.666	0.008
SE	-0.037	0.012	-3.157	0.002

Notes: PO – Power, AC – achievement, SD – self-direction, UN – universalism, BE – benevolence, CO – conformity, TR – tradition, SE – security.

Table A.12: Ordinary least squares regression results across European welfare state regimes

	**netinum**	**netinum**	**netinum**	**netinum**	**netinum**
Predictors	*Estimates*	*Estimates*	*Estimates*	*Estimates*	*Estimates*
(Intercept)	-0.240 ***	0.014	0.001	-0.128 ***	-0.070 ***
agea demean	1.444 ***	1.371 ***	1.440 ***	1.000 ***	0.337 **
agea 2 demean	-11.375 ***	-11.422 ***	-13.906 ***	-7.290 ***	-2.909 **
PO	0.062 .	0.083 ***	0.032	-0.015	0.002
AC	-0.008	0.031 .	-0.016	-0.026	0.031 .
SD	-0.061	0.025	-0.036	0.025	0.069 **
UN	0.079 .	0.036	-0.037	-0.054	-0.023
BE	-0.041	0.007	-0.087 .	-0.063 .	0.021
CO	-0.030	-0.005	-0.033	-0.004	0.002
TR	-0.066 *	-0.036 .	-0.025	0.029	-0.083 ***
SE	-0.057 .	-0.037 *	-0.030	-0.050 .	-0.019 *
Observations	1382	4716	2430	1569	6338
R^2 / R^2 adjusted	0.104 / 0.097	0.195 / 0.193	0.129 / 0.125	0.071 / 0.065	0.058 / 0.056

*. p<0.1 * p<0.05 ** p<0.01 *** p<0.001*

Notes: PO – Power, AC – achievement, SD – self-direction, UN – universalism, BE – benevolence, CO – conformity, TR – tradition, SE – security.

Additional references

102. Card, D. (1999). The Causal Effect of Education on Earnings. *Handbook of Labor Economics*, 3, 1801-1863.
103. Wooldridge, J. M. (2010). *Econometric Analysis of Cross Section and Panel Data.* Cambridge, MA: MIT Press.
104. Fox, J. and Weisberg, S. (2019) An R Companion to Applied Regression, Third Edition, Sage.
105. Hebbali A (2020). *olsrr*: Tools for Building OLS Regression Models_. R package version 0.5.3, https://CRAN.R-project.org/package=olsrr.
106. Cook, R. D.; Weisberg, S. (1983). Diagnostics for Heteroskedasticity in Regression. *Biometrika*. 70 (1): 1–10.
107. White H. (1980). A Heteroskedasticity-Consistent Covariance Matrix and a Direct Test for Heteroskedasticity. *Econometrica* 48, 817–838.
108. Sargan, J. D. (1958). "The Estimation of Economic Relationships Using Instrumental Variables". *Econometrica*, 26 (3): 393–415.
